# Human Gut Microbiome-Based Knowledgebase as a Biomarker Screening Tool to Improve the Predicted Probability for Colorectal Cancer

**DOI:** 10.3389/fmicb.2020.596027

**Published:** 2020-11-19

**Authors:** Zhongkun Zhou, Shiqiang Ge, Yang Li, Wantong Ma, Yuheng Liu, Shujian Hu, Rentao Zhang, Yunhao Ma, Kangjia Du, Ashikujaman Syed, Peng Chen

**Affiliations:** ^1^School of Pharmacy, Lanzhou University, Lanzhou, China; ^2^Department of Electronic Information Engineering, Lanzhou Vocational Technical College, Lanzhou, China

**Keywords:** biomarkers, colorectal cancer, database, diagnosis, microbiome

## Abstract

Colorectal cancer (CRC) is a common clinical malignancy globally ranked as the fourth leading cause of cancer mortality. Some microbes are known to contribute to adenoma-carcinoma transition and possess diagnostic potential. Advances in high-throughput sequencing technology and functional studies have provided significant insights into the landscape of the gut microbiome and the fundamental roles of its components in carcinogenesis. Integration of scattered knowledge is highly beneficial for future progress. In this study, literature review and information extraction were performed, with the aim of integrating the available data resources and facilitating comparative research. A knowledgebase of the human CRC microbiome was compiled to facilitate understanding of diagnosis, and the global signatures of CRC microbes, sample types, algorithms, differential microorganisms and various panels of markers plus their diagnostic performance were evaluated based on statistical and phylogenetic analyses. Additionally, prospects about current changelings and solution strategies were outlined for identifying future research directions. This type of data integration strategy presents an effective platform for inquiry and comparison of relevant information, providing a tool for further study about CRC-related microbes and exploration of factors promoting clinical transformation (available at: http://gsbios.com/index/experimental/dts_ mben?id=1).

## Introduction

Colorectal cancer (CRC) is a common malignancy worldwide accounting for about 1 in 10 cancer cases, with incidence and mortality rates of 6.1 and 9.2%, respectively (Bray et al., 2018). Various genetic and environmental factors contribute to CRC development from aberrant crypts to tumors. Overall, ∼3 × 10^13^ bacteria colonize the human gut and abnormal microbiome composition has been shown to contribute to the initiation, progression and metastasis of CRC (Pitot, 1993; Qin et al., 2010; Wong et al., 2017c). In cases where patients are rapidly diagnosed and treated with surgery at the early stages, survival exceeds 90%. However, the survival rate is significantly decreased to 13% in patients with advanced metastatic disease (Shah et al., 2018). The potential value of microorganisms in early diagnosis has attracted significant research attention over the last few decades.

The term “microbiome” refers to the entire habitat including microorganisms (bacteria, archaea, lower and higher eukaryotes, and viruses), their genomes, and the surrounding environmental conditions (Marchesi and Ravel, 2015). These factors are altered along the adenoma-carcinoma sequence, reflected by changes in abundance. Some microbes produce genotoxic compounds and induce inflammation while others proliferate in the tumor-associated niche, designated “driver” and “passenger” bacteria, respectively (Tjalsma et al., 2012). Systematic analysis of microbial communities and identification of those with differential abundance as biomarkers presents an effective diagnostic strategy. Further advances, such as next-generation sequencing, have generated massive amounts of data on the CRC microbiome. Bioinformatics as well as machine learning methods additionally provide powerful tools to advance our understanding (Tabib et al., 2020). Metagenomics and 16S rRNA sequencing studies have revealed different abundance of some microbes between patients and healthy populations and effective combinations of microbial biomarkers could be applied for CRC diagnosis (Sze and Schloss, 2018; Thomas et al., 2019b). Upon combination of these strategies with the fecal immunochemical test (FIT), superior sensitivity and area under the receiver operating characteristic curve (AUC) were obtained relative to standalone FIT, which facilitated advanced adenoma detection (Wong et al., 2017a). Several microbes have been linked with CRC development, including *Fusobacterium nucleatum* (Fn), *Peptostreptococcus anaerobius* (Pa), *Parvimonas micra* (Pm), *Enterotoxigenic Bacteroides fragilis* (ETBF), *Peptostreptococcus stomatis* (Ps) and *Escherichia coli* (Yu et al., 2017a; Pleguezuelos-Manzano et al., 2020). Recently, the ratio of pathogenic bacteria to probiotic populations with decreased abundance in CRC patients was used in a diagnostic model based on their antagonistic effect (Guo et al., 2018). Metabolomics and metagenomics studies have shown that shifts in pathogenicity island genes, short-chain fatty acids (SCFA), amino acids, butyrate and bile acids occur at the early stages of CRC development. Some of these factors possess health-promoting and antineoplastic properties, such as maintenance of mucosal integrity and suppression of inflammation and carcinogenesis. Thus, the shift, particularly the decrease of these health-promoting factors, could contribute to the malignant outgrowth of the tumors (O’Keefe, 2016; Yachida et al., 2019). Subsequent mechanistic research further confirmed their involvement in CRC. For instance, Fn harbors the FadA virulence factor, which binds E-cadherin and activates Wnt/β-catenin and TLR4/MYD88 pathways to promote cancer initiation, proliferation and invasion (Rubinstein et al., 2013, 2019). Enterotoxigenic *Bacteroides fragilis*(ETBF) harbors the toxin BFT that causes inflammatory diarrhea, inflammation-related tumorigenesis and upregulation of spermine oxidase. Colibactin-producing *E. coli* alkylates DNA at adenine residues and induces double-stranded breaks, anaphase bridges and chromosome aberrations (Cuevas-Ramos et al., 2010; Goodwin et al., 2011; Chung et al., 2018; Pleguezuelos-Manzano et al., 2020). Based on these omics and experimental data, a theoretical foundation for clinical translation was proposed, which requires validation with more economical methods, such as quantitative PCR (qPCR), or integration with other indices, such as FIT, to obtain optimal benefits (Wong et al., 2017a). More novel biomarkers should emerge with further research progress. However, effective diagnostic panels remain to be established.

While several meta-analyses and reviews based on large-scale, cross-cohort studies have revealed robust associations between microbiome and diseases, developing solutions from the perspective of integration remains a considerable problem due to a number of reasons. First, among the published studies, feces is the most common sample type owing to the non-invasive nature and convenience of sample collection. Other non-invasive types of samples, such as oral swabs, offer an alternative but still need more studies (Flemer et al., 2018). Second, a number of studies were based on 16S rRNA sequencing while others involved metagenomics analyses, which may generate different taxonomic resolutions and involve distinct bioinformatics methods (Wirbel et al., 2019). Third, robustness among different countries or regions is another key contributory factor in microbiome composition, including genetic background, dietary habits and the environment. Fourth, optimal numbers of microbial markers recorded are significantly variable among studies (Duvallet et al., 2017). Fifth, specificity deserves further research attention, since only a few studies to date have included cases of other diseases. For example, *Helicobacter pylori* and human papillomavirus are specifically associated with gastric and cervical cancer types while other microbes, such as the order of *Clostridiales* (*Lachnospiraceae* and *Ruminococcaceae* families), are non-specifically associated with disease (Duvallet et al., 2017). In general, integration of different types of markers may obtain higher sensitivity, yet specificity will decrease. Therefore, biomarkers that are specific to CRC are of great importance. Finally, classification basis, algorithms, costs and standardization are also worth noting, but systematic integration of the data is lacking.

In this study, a knowledgebase of CRC-related microbes was established by reviewing the relevant literature and extracting key information. Next, a web-based platform using structured query language (SQL) was constructed and statistical analysis were performed that included three classifications and more than seven hundred records of microbial markers. By integrating the scattered data, our novel database could be used to perform inquiry and comparison across different models or databases, such as SILVA, VFDB and the Human Microbiome Oral Database (HOMD), thus contributing to the study of microbiome-based diagnosis of CRC.

## Materials and Methods

### Database Construction

Literature was retrieved from PubMed during September 2019 and April 2020 based on the relevant search criteria. Two keyword groups were used, the first being “colorectal cancer” and second comprising “16S rDNA,” “metagenomics,” “sequencing,” “quantitative real-time PCR,” “biomarker,” “diagnosis,” “screening,” and “microbiome.” Studies that used blood samples or focused on prognosis, genes, methylation, proteins, small molecule metabolites and liquid biopsy biomarkers were excluded. Following a comprehensive search of the literature and supplementary materials, the relevant data, including names of microbes, sensitivity, specificity, changes in abundance, functions of microbes, technology, algorithm, number of cases, sources and links, were collected. Furthermore, information of the taxonomy of microbial markers was collected from NCBI (Taxonomy) and added into the database. Ultimately, biomarkers were classified into three categories. Microbes that displayed statistical significance in both high-throughput sequencing/pyrosequencing and qPCR experiments were defined as “Class One,” those confirmed with one of the above techniques as “Class Two,” and combinations of different microbes for diagnosis as “Class Three.” Notably, these candidates specifically refer to gut bacteria although the gut microbiome comprises bacteria, fungi, archaea, viruses and bacteriophages.

### Data Query and Display

**FIGURE 1:**
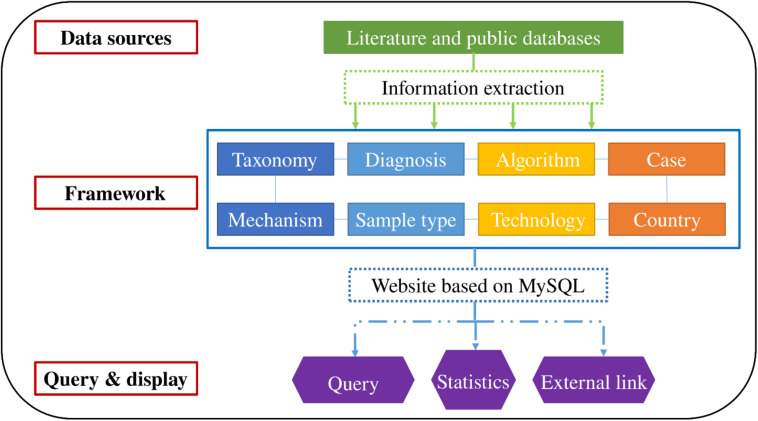
Construction and framework of the database.

Integrated data were accessible through a web interface that indirectly generates MySQL queries. The interface supports query functions, such as “scientific name of the bacterium” and “taxonomy.” Additionally, basic statistics and visualization were performed according to personalized requirements. Article links for verification or further research are provided for interested authors. The organizational framework is presented in Figure 1.

### Construction of the Phylogenetic Tree and Statistical Analysis of CRC-Associated Microbes

16S rRNA sequences of all the species (all CRC-associated overabundant and depleted species) in the database were aligned using MEGA-X v10.1.8 software (Kumar et al., 2018). Phylogenetic tree was constructed using the following settings: maximum likelihood as the statistical method, 500 bootstrap replications, Kimura two-parameter as the substitution model and Near-Neighbor-Interchange as the ML Heuristic method. Finally, the tree was adjusted and visualized in Interactive Tree Of Life (iTOL)^1^ (Letunic and Bork, 2019). Other statistical analyses were performed with OriginPro software (OriginLab Corporation, United States).

## Results and Discussion

### Global Signature of CRC-Related Microbes

**FIGURE 2:**
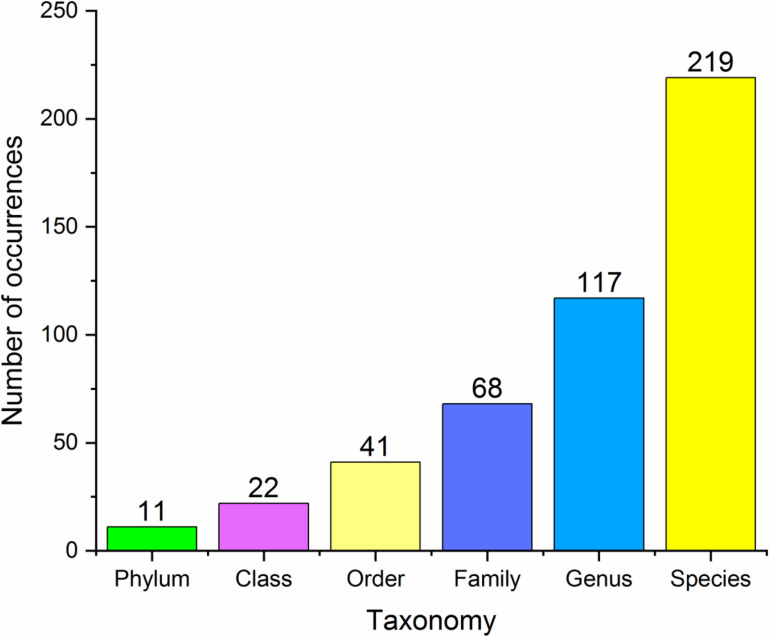
Basic statistics at different taxonomy levels of all the microbial markers in the database.

In our database, 17 species belonged to Class One (microbes with statistical importance verified using both high-throughput sequencing/pyrosequencing and qPCR), 219 species/clusters to Class Two (microbes confirmed via high-throughput sequencing/pyrosequencing or qPCR), including 11 phyla, 22 classes, 41 orders, 68 families and 117 genera (Figure 2), and 41 panels to Class Three (combinations of different microbes for diagnosis). Despite many microbes proposed for diagnosis and several confirmed conclusions, inconsistent results have been obtained by different research groups.

In healthy individuals, the most dominant phyla (over 90%) are *Firmicutes*, *Bacteroidetes, Proteobacteria* and *Verrucomicrobia* (Eckburg et al., 2005). Moreover, significant differences between healthy individuals and CRC patients are detected. Meanwhile, these differences of indices usually showed stepwise decreased or increased frequency from controls, to dysplasia to cancers, though some changes may not be statistically significant between healthy and adenoma groups. In addition to relative abundance, differences in other indices, such as alpha and beta diversity, have been identified. Feces of healthy controls generally contain microbial communities with higher diversity while tissue samples from CRC patients show greater alpha diversity. Earlier studies revealed greater microbial diversity in tumor samples compared with control and polyp samples, with a 75% higher estimated number of species than tissues from healthy sites (Mira-Pascual et al., 2015; Vogtmann et al., 2016), characterized by increased levels of opportunistic pathogens. Chao1 and Shannon indices are commonly used to estimate microbial richness and diversity. Decreased Shannon and Chao1 indices were recently reported in fecal samples collected from CRC patients (Yang et al., 2019). Similarly, in an azoxymethane (AOM) mouse model, the CRC group showed significantly lower bacterial richness and Shannon-Weaver’s diversity index (Wong et al., 2017b). Other analyses revealed no significant differences in either richness or biodiversity, which could be attributable to the relatively small study cohorts (Wu et al., 2013; Youssef et al., 2018). However, differences at the taxonomic levels (family, genus and species) were universally observed. For instance, patients with CRC usually have increased abundance of operational taxonomic units (OTU) assigned as *Ruminococcus*, *Porphyromonas*, *Peptostreptococcus*, *Parvimonas*, and *Fusobacterium*, while healthy individuals possess more beneficial butyrate-producing bacteria, such as *Bifidobacterium* and *Clostridium butyricum* (Flemer et al., 2017; Sacks et al., 2018). The collective results clearly demonstrate differences in microbial populations between CRC and healthy groups.

### Biomarker Identification for Diagnosis

#### Sample Types Used for Diagnosis

In studies on CRC-related microbes, fecal samples from CRC and adenoma patients and healthy volunteers were the most commonly used owing to the non-invasive nature and convenience of sample collection. Cancerous and adjacent non-cancerous normal tissues represent another type of sample that can effectively reveal the overall structure of microbiota in the tumor microenvironment but are unsuitable for early diagnosis (Gao et al., 2015). The microbial diversity in fecal samples is twice as high as that in tissue samples (Mira-Pascual et al., 2015). Oral swabs represent another novel sample type. Previously identified biomarkers, such as *Fusobacterium nucleatum* and *Parvimonas micra*, are oral microbes. An earlier investigation profiled the oral microbiome as an alternative screening method for CRC (Flemer et al., 2018). Interestingly, a retrospective study on data obtained from adult patients diagnosed with bacteremia and subsequently CRC reported association with *Bacteroides fragilis*, *Streptococcus gallolyticus* and other intestinal microbes, thus providing a new perspective for clinicians (Kwong et al., 2018). Recently, (Poore et al., 2020) reported that predictions based on microbial DNA in blood could discriminate CRC from healthy, cancer-free individuals. However, blood samples were not included in this database due to the requirement for further exploration.

#### Diagnostic Techniques

This database involves five technical protocols, specifically, denaturing gradient gel electrophoresis (DGGE), qPCR, pyrosequencing, 16S rRNA sequencing and metagenomics sequencing, which have various advantages and disadvantages. Initially, the culture-dependent method was used to analyze CRC microbes as early as the 1960s, which led to significant underestimation of microbial diversity (Wong and Yu, 2019). Recently, a library containing 7,758 human gut bacterial isolates was constructed. Although culture-based methodologies provide access to data that both overlap and complement sequencing surveys, yet these protocols were both labor- and time-consuming compared with culture-independent methods (Poyet et al., 2019). Molecular analysis technology has developed from DGGE and qPCR to high-throughput sequencing over the years. While the efficiency of analysis was improved by DGGE and qPCR, limitations of low throughput remained unresolved. In 2005, the introduction of next-generation sequencing (NGS) facilitated massive parallel, low-cost and rapid sequencing. 16S rRNA and metagenomics sequencing have further improved efficiency and are widely employed at present. The former procedure is based on the 16S rRNA gene amplicon and facilitates taxonomic and phylogenetic analyses. While the cost-effective feature enables its universal application, several limitations exist: (1) amplicon sequencing of 16S rRNA gene via PCR may miss OTU/taxa detection due to various biases associated with PCR, (2) possible overestimation of community diversity or species abundance, and (3) lack of ability to directly analyze the biological functions of associated taxa (Xia et al., 2018). Recently, potentially unbiased shotgun metagenomics analyses have been conducted, which provide higher taxonomic resolution, gene function and comparative analyses at a decreased cost (Wirbel et al., 2019). However, in terms of clinical transformation, the qPCR-based method is more economical and rapid.

#### Algorithms Used for Diagnosis

Algorithms include the processes of classification, biomarker identification and model prediction. The classification approaches comprise OTU-based, metagenomics linkage group (MLG)-based, integrated microbial genome (IMG)-based and co-abundant gene group (CAG)-based methods. The model prediction algorithms include random forest (RF), support vector machine (SVM), logistic regression (LR) and leave-one-dataset-out (LODO) analyses, among which random forest is the most widely used algorithm. For the biomarker identification process, relative abundance and Linear discriminant analysis Effect Size (LEfSe) methods are the most commonly used.

Random forest provides a measure of variable importance and out-of-bag (OOB) error when building a tree, making it suitable for prediction analysis. A recent meta-analysis employed the random forest classifier to determine accurate predictive models using a minimal microbial signature. The data showed that using 16 species, cross-validation of AUC > 0.80 was achieved for the majority of datasets (Thomas et al., 2019a). SVM is advantageous for classifying small data volumes and achieved an overall AUC of 0.80 for the combined population (Dai et al., 2018). Recent studies have examined different machine leaning classifiers, including RF, Bayesian network, SVM, k-Nearest neighbor and general regression neural networks (Arabameri et al., 2020). LR, applied by most studies, is used to predict binary outcome from a set of numeric variables and aims to identify the most significant features (Wong et al., 2017a). Phylotype-based and OTU-based methods are the main approaches for sequence identification, with the latter being most widely used. However, the OTU-based method has a number of limitations, such as a computationally intensive protocol and larger memory requirement (Schloss and Westcott, 2011). Other methods have been developed to overcome these drawbacks. For instance, CAGs have been proposed to mitigate the ultrahigh dimensionality challenge of gene-level metagenomics (Minot and Willis, 2019). In addition, CAG-based clusters could be used to determine CRC-associated microbe profiles (Flemer et al., 2017). Taking the collective factors (such as data quantity, number of cohorts and risk factors) into consideration, appropriate approaches and classifiers should be adopted.

#### Overview of Current Biomarkers for Diagnosis

More than 200 species belonged to the Class Two microbe group (confirmed using either high-throughput sequencing/pyrosequencing or qPCR), among which only 17 were verified as statistically significant with both high-throughput sequencing/pyrosequencing and qPCR (Class One). Fn is a known opportunistic pathogen showing increased abundance in feces of CRC patients with a sensitivity range of 69.2–82.9%, specificity of 52.8–90.8% and AUC of 0.675–0.875. Combined with FIT or fecal occult blood test (FOBT), sensitivity, specificity and AUC values reached 92.3, 94.4% and 0.95, respectively. Recently, a number of novel markers have been shown to perform well in CRC diagnosis. Pa was increased in four different cohorts and induced carcinogenesis in mice via a PCWBR2-integrin α2/β1-PI3K–Akt–NF-κB signaling axis with a sensitivity of 79.8% and specificity of 98% in combination with FIT (Yu et al., 2017a; Long et al., 2019). *Lachnoclostridium sp.* (designated m3) sharing 97% (1883/1935) DNA sequence similarity with *Lachnoclostridium sp.* YL32 was significantly enriched in adenoma. m3 showed specificity of 78.5% and sensitivity of 48.3% for adenoma and 62.1% for CRC. However, its role in tumorigenesis warrants further research (Liang et al., 2019). The other 15 biomarkers are presented in Table 1 (4 were decreased and 11 were enriched in patients).

**TABLE 1:** Diagnostic performance of Class One microbials.

Name	Sensitivity%	Specificity%	AUC	Algorithm	Sample	Case	Region	References
Fn	73.1	90.8	0.860	Relative Abundance	Feces	490	China	Wong et al., 2017a
Pa	56.7	86.3	0.720	Logistic regression	Feces	390	China	Wong et al., 2017a
Pm	45.2	97.1	0.730	Logistic regression	Feces	390	China	Wong et al., 2017a
Gm	39.0	76.0	0.622	Relative Abundance	Feces	333	Spain	Malagón et al., 2019
Ps	53.0	76.0	0.710	Relative Abundance	Feces	333	Spain	Malagón et al., 2019
Bf	33.0	0.76	0.571	Relative Abundance	Feces	333	Spain	Malagón et al., 2019
pks	56.4	82.0	NA	Relative Abundance	Feces	238	Sweden	Eklöf et al., 2017
Fp	81.8	62.6	0.741	Abundance Rate	Feces	549	China	Rezasoltani et al., 2018
Bb	90.4	76.4	0.870	Abundance Rate	Feces	549	China	Rezasoltani et al., 2018
Cs	73.3	66.1	0.736	logistic regression	Feces	781	China	Xie et al., 2017
Ap	NA	NA	NA	Relative abundance	Feces	146	Meta	Yachida et al., 2019
Gl	NA	NA	NA	Relative abundance	Mucosa	207	China	Nakatsu et al., 2015
m3	62.1	79.0	0.741	Relative Abundance	Feces	1012	China	Liang et al., 2019
Bd	NA	NA	NA	Relative abundance	Feces	179	French	Sobhani et al., 2011
afaC	NA	NA	NA	Relative abundance	Tissue	55	South Africa	Viljoen et al., 2015
Akk	NA	NA	NA	Relative abundance	Feces	112	China	Wang et al., 2020
Cb	NA	NA	0.930	Random forest	Feces	60	China	Yang et al., 2020

*NA, non-available; Meta, meta-analysis; Gemella morbillorum (Gm); Bacteroides fragilis (Bf); pks + clbA + Escherichia coli (pks); Clostridium symbiosum (Cs); Atopobium parvulum (Ap); Granulicatella (Gl); Bacteroides (Bd); afaC-positive E. coli (afaC).*

**FIGURE 3:**
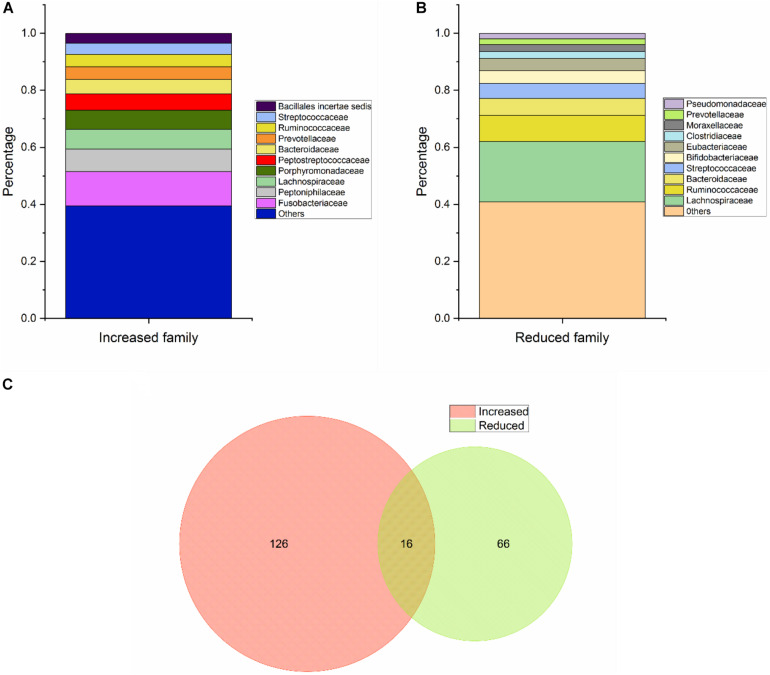
Basic statistical analysis of Class Two microbes (shown to be significant via high-throughput sequencing/pyrosequencing or qPCR) in the database. **(A)** Statistical analysis of the top 10 increased microbes at the family level. **(B)** Statistical analysis of the top 10 reduced microbes at the family level. **(C)** Venn diagram of all CRC-associated microbes at the species level.

**FIGURE 4:**
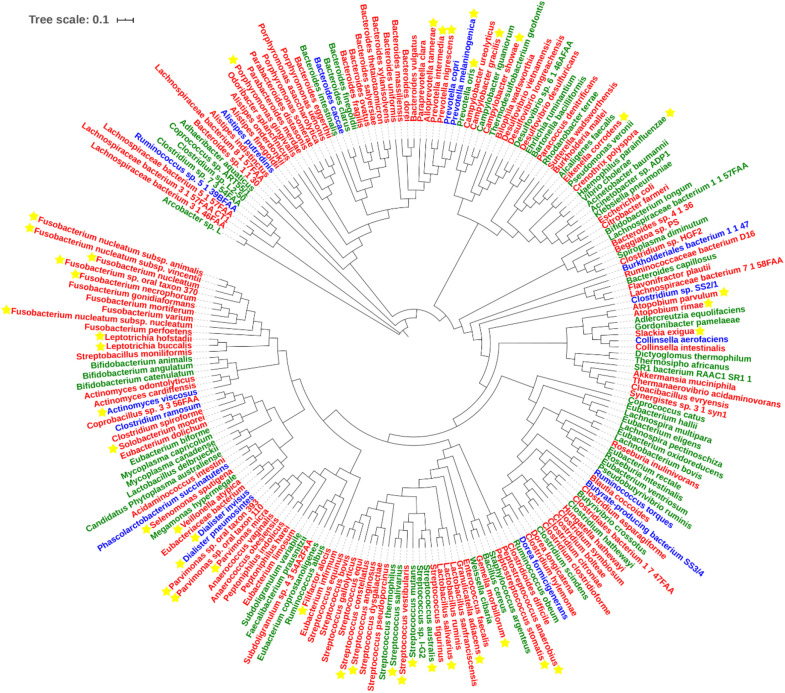
Phylogenetic tree of all CRC-related microbes in the database. Species marked in red and green refer to the increased and decreased microbes, and species marked in blue refer to the microbes that show up in both increased and decreased groups. Species marked with yellow stars refer to oral microbes according to HMOD (16S rRNA sequences of m7 and *Sulfurovum* sp. SCGC AAA036-O23 are not available, which also belong to the increased group).

With regard to Class Two microbes, basic statistics are shown in Figure 3 and phylogenetic tree in Figure 4. The majority of enriched microbes were classified into *Fusobacteriaceae*, *Peptoniphilaceae*, *Lachnospiraceae*, *Porphyromonadaceae*, *Peptostreptococcaceae*, *Bacteroidaceae*, *Prevotellaceae, Ruminococcaceae, Streptococcaceae*, and *Bacillales incertae sedis* at the family level (Figure 3A). Among the group of decreased microbes, most were classified into *Lachnospiraceae*, *Ruminococcaceae*, *Bacteroidaceae, Streptococcaceae, Bifidobacteriaceae*, and *Eubacteriaceae* (Figure 3B). In the Venn diagram, only a small overlap of increased and decreased microbes was observed, supporting the reliability of most microbial markers despite some inconsistencies (Figure 3C). At the species level, phylogenetic tree showed details of current CRC-related biomarkers as well as their evolutionary relationships. Additionally, species belonging to oral microbes were marked with stars.

The functions of gut microbes include fermenting complex carbohydrates to produce large amounts of metabolites, maintaining epithelial homeostasis, serving as an endocrine organ and participating in the development, maturation and differentiation of the immune system of the host (Villéger et al., 2018; Rastelli et al., 2019). In a sense, intestinal metabolites directly affect the occurrence of CRC and not intestinal flora. The majority of nutrients from food are absorbed in the small intestine with protein residues and complex nutrients, such as fiber moving to the colon, and consequently metabolized by the microbial populations (O’Keefe, 2016). Therefore, from the perspective of microbial function, the majority are associated with protein fermentation, bile acid biotransformation, decomposition of polysaccharides and polyphenols and energy metabolism. For example, *Faecalibacterium prausnitzii* (Fp), *Bifidobacterium* (Bb), *Roseburia* spp. (Rb), *Eubacterium rectale* (EUB), *Clostridium butyicum* (Cb), *Lactobacillus* spp. (Lc), *Akkermansia muciniphila* (Akk), *Ruminococcus*, and *Lachnospiraceae* were found to be more abundant in healthy controls compared with CRC patients. Fp is a butyrate producer decreased in Crohn’s disease (CD) patients, whose metabolites exert anti-inflammatory effects via blocking NF-κB activation and IL-8 production (Sokol et al., 2008). Bb and Lc are used as probiotics for human consumption and benefit the gut through inducing cancer cell apoptosis, inhibiting cell proliferation, modulating host immunity and inactivating carcinogenic toxins (Wong and Yu, 2019). An earlier study reported that determination of Fn/Bb and Fn/Fp ratios could improve diagnostic performance for CRC based on their antagonistic effect (Rezasoltani et al., 2018). Both Rb and EUB are butyrate-producing *Firmicutes* and metabolize dietary fibers to provide energy sources and achieve anti-inflammatory effects (Paramsothy et al., 2019). Their capabilities as a non-invasive tool were additionally evaluated but not included in the final model (Malagón et al., 2019). More recently, the utility of other widely recognized markers, including Fn, colibactin-producing *E. coli* and ETBF, in diagnosis of CRC has been systematically analyzed (Chung et al., 2018; Malagón et al., 2019; Wu et al., 2019; Pleguezuelos-Manzano et al., 2020). However, several issues require further clarification. Although the pathogenesis and benefits of ETBF and Bb have been validated, inconsistencies exist among different samples. ETBF was shown to be increased in tumor tissues and form a biofilm in the gut. However, this pathogenic bacterium displayed no significant differences in abundance in patient fecal samples and was not detectable using qPCR targeting the toxin-producing gene, making it difficult to discriminate between patients and healthy controls (Zackular et al., 2014; Kosumi et al., 2018; Sze and Schloss, 2018; Malagón et al., 2019; Saffarian et al., 2019). Finally, *Lachnospiraceae* and *Ruminococcaceae* families were associated with multiple diseases (known as non-specific responders), which inspired us to obtain non-gastrointestinal cancer samples for future experimental design (Duvallet et al., 2017; Rezasoltani et al., 2018).

### Diagnostic Strategy and Performance

#### Combinations of Different Microbial Markers

Class Three (combinations of different microbes for diagnosis) included 41 panels verified using various methods (Table 2). The combinations ranged from two species to 63 OTUs, with AUC ranging from 0.531 to 0.998. Twelve panels were based on qPCR, whose algorithms usually link with logistic regression or relative abundance. Meanwhile, 16 panels and 12 combinations were based on 16S rRNA and metagenomics sequencing data, predominantly using the random forest-based model. Based on AUC, qPCR-based models could achieve comparable outcomes to the two other technologies with limited biomarkers (usually no more five species). Nevertheless, 16S rRNA and metagenomics-based models show performance advantages at the cost of the number of markers (more than 10 OTUs on average). In the random forest and Minimum Redundancy Maximum Relevance (mRMR) models, both OOB and error rate parameters demonstrated that panels comprising ∼16–20 biomarkers achieved the best prediction accuracy (Flemer et al., 2018; Wirbel et al., 2019).

**TABLE 2:** Different panels for CRC screening.

Name	Sensitivity%	Specificity%	AUC	Technique	Algorithm	Sample	Case	Region	References
Fn, Pa, Pm	89.4	93.0	0.950	qPCR	LR	Feces	390	China	Wong et al., 2017a
Ps/EUB, Bf/EUB, Bt/EUB	80.0	90.0	0.837	qPCR	LR	Feces	333	Spain	Malagón et al., 2019
pks, Fn	89.7	61.0	NA	qPCR	DA	Feces	238	Sweden	Eklöf et al., 2017
Fn/Fp	95.0	71.3	0.914	qPCR	AR	Feces	549	China	Rezasoltani et al., 2018
Fn/Bb	84.6	92.3	0.911	qPCR	AR	Feces	549	China	Rezasoltani et al., 2018
Fn/Fp, Fn/Bb	80.8	85.6	0.910	qPCR	AR	Feces	549	China	Rezasoltani et al., 2018
Fn, Fp, Bb	92.5	83.5	0.943	qPCR	AR	Feces	549	China	Rezasoltani et al., 2018
5 OTUs	90.0	80.0	0.896	16SrDNA	LR	Feces	90	America	Zackular et al., 2014
6 OTUs	90.0	83.0	0.922	16SrDNA	LR	Feces	90	America	Zackular et al., 2014
22OTUs	81.2	97.1	0.673	16SrDNA	RF	Feces	490	Canada, United States	Baxter et al., 2016b
34 OTUs	51.7	97.1	0.847	16SrDNA	RF	Feces	490	Canada, United States	Baxter et al., 2016b
23 OTUs	70.0	92.8	0.829	16SrDNA	RF	Feces	490	Canada, United States	Baxter et al., 2016b
16 OTUs	53.0	96.0	0.900	16SrDNA	RF	Oral swabs	60	Ireland	Flemer et al., 2018
28 OTUs (16 oral swabs, 12 feces)	74.0	94.0	0.940	16SrDNA	RF	Feces and oral swabs	60	Ireland	Flemer et al., 2018
63 OTUs (29 oral swabs, 34 feces)	88.0	94.0	0.980	16SrDNA	RF	Feces and oral swabs	60	Ireland	Flemer et al., 2018
22 OTUs	58.0	92.0	0.840	Metagenomics	LR	Feces	156	France, Germany	Zeller et al., 2014
7 OTUs	87.0	83.7	0.886	Metagenomics	RF	Feces	128	China	Yu et al., 2017a
15 MLGs	NA	NA	0.983	Metagenomics	RF	Feces	96	Austria	Feng et al., 2015
16 OTUs	NA	NA	0.860	Metagenomics	RF	Feces	969	Meta	Thomas et al., 2019a
17 OTUs	60.1	84.8	0.804	Metagenomics	RF	Feces	424	Meta	Shah et al., 2018
30 OTUs	NA	NA	0.830	Metagenomics	RF	Feces	208	Meta	Yachida et al., 2019
8 taxa	NA	NA	0.750	16SrDNA	RF	Feces	492	Meta	Yachida et al., 2019
12 genus	NA	NA	0.846	16SrDNA	RF	Feces	1674	Meta	Sze and Schloss, 2018
18 OTUs	NA	NA	0.831	16SrDNA	RF	Feces	404	Canada, United States	Baxter et al., 2016a
32 OTUs	NA	NA	0.853	16SrDNA	RF	Feces	404	Canada, United States	Baxter et al., 2016a
41 OTUs	NA	NA	0.686	16SrDNA	RF	Feces	404	Canada, United States	Baxter et al., 2016a
12 phylotypes	NA	NA	0.831	16SrDNA	LEfSe	Mucosa	160	China	Nakatsu et al., 2015
18 OTUs	NA	NA	0.871	16SrDNA	RF	Mucosa	160	China	Nakatsu et al., 2015
38 phylotypes	NA	NA	0.846	16SrDNA	Dirichlet MM	Mucosa	160	China	Nakatsu et al., 2015
m3, Fn, Ch, Bc	85.2	80.2	0.907	qPCR	LR	Feces	1012	China	Liang et al., 2019
m3, Fn	NA	NA	0.891	qPCR	LR	Feces	1012	China	Liang et al., 2019
Fn, Ch, m7, Bc	92.8	79.8	0.886	qPCR	SLC	Feces	370	China	Liang et al., 2017
Fn, Ch, m7, Bc, Ri	74.3	88.9	0.843	qPCR	LR	Feces	128	China	Liang et al., 2017
17 IMG species	NA	NA	0.860	Metagenomics	IMG	Feces	128	China	Liang et al., 2017
7 species-level mOTUs	NA	NA	0.890	Metagenomics	mOTUs	Feces	128	China	Liang et al., 2017
27 MLG	NA	NA	0.960	Metagenomics	MLG	Feces	128	China	Liang et al., 2017
Fn, Pa, Pm (4 genes)	NA	NA	0.770	Metagenomics	CRC index	Feces	96	China, Denmark, Austrian, French	Yu et al., 2017a
22 genes	NA	NA	0.998	Metagenomics	RF	Feces	107	China	Yang et al., 2020
Cb, Cs	NA	NA	0.935	qPCR	RF	Feces	60	China	Yang et al., 2020
7 CRC-enriched bacteria	NA	NA	0.800	Metagenomics	SVM	Feces	526	Meta	Dai et al., 2018
55 species	NA	NA	0.830	Metagenomics	RF	Feces	181	Meta	Sze and Schloss, 2018

*DA, Decision Abundance; AR, Abundance Rate; SLC, Simple linear combination; Dirichlet MM, Dirichlet multinomial mixtures; Clostridium hathewayi (Ch); Unclassified species (m7); Roseburia intestinalis (Ri); Bacteroides thetaiotaomicron (Bt).*

Combination of microbes may be operative, rather than representing a strain that is increased or decreased in the intestine (Tilg et al., 2018). In addition, prediction models from single dataset may lead to reduced accuracy and be sensitive to both technique and heterogeneity (Thomas et al., 2019a). An earlier study identified 63 OTUs (29 from oral swabs and 34 from fecal samples) to predict CRC. While the final AUC value was up to 0.98, its application in clinical examination remains a challenge (Flemer et al., 2018). Several other researchers used more than 30 OTUs/phylotypes/MLGs to construct a random forest classifier and obtained AUC values >0.80 (Nakatsu et al., 2015; Baxter et al., 2016a; Yu et al., 2017a). Previous studies suggest that the *Firmicutes*/*Bacteroidetes* ratio responds to health and disease states, such as obesity and CRC (Ley et al., 2006; Saffarian et al., 2019). Interactions between bacteria provide an ecological perspective for screening, and increase in pathogenic bacteria is always accompanied by decrease in beneficial microbes (Dai et al., 2018). Some researchers observed an association of the group of *Bacteroides* and *Prevotella* with elevated IL17-producing cells in colon cancer and demonstrated that supernatant from Fn inhibited the bactericidal activities of Fp and Bb (Sobhani et al., 2011; Guo et al., 2018). Furthermore, beneficial microbes can contribute to several intestinal functions and protect the organ from pathogenic microorganisms, and the “pathogenic bacteria:probiotics” ratio generates a better effect than single organism model (Eslami et al., 2019; Malagón et al., 2019; Yang et al., 2020). Thus, the complementary effects between enriched and reduced microbes should be highlighted for further investigation. Clearly, combinations of different microbial markers exhibit better predictive performance than single markers.

#### Integration With FIT

In the database, FIT was also presented when available. FIT has been extensively tested and recommended by National Comprehensive Cancer Network guidelines. The method involves direct detection of globin rather than heme, and shows greater sensitivity than the highly sensitive guaiac fecal occult blood test. Retrospective analysis showed that replacing 3-year colonoscopy surveillance with annual FIT could reduce the requirement for colonoscopy and provide economic benefits. However, sensitivity was relatively low for advanced neoplasms, ranging from 21.8 to 46.3% at the preset thresholds (Gies et al., 2018; Cross et al., 2019a). Combining microbe analysis with FIT could enhance the detection of advanced precancerous lesions, as validated in numerous experiments. Taking results from Class One and Three as representative cases, combined quantitation of Fn and FIT showed superior sensitivity to FIT alone, leading to detection of lesions missed by FIT alone (Wong et al., 2017a). Similarly, Pa, Pm, Cs, and m3 displayed an obvious improvement in both sensitivity and AUC, with a slight decrease in specificity (Xie et al., 2017; Liang et al., 2019). This complementary role was also illustrated using biomarker panels. Upon combining 22 OTUs identified using the penalized linear model with FIT, sensitivity increased from 58 to 72% at the same specificity (Zeller et al., 2014). In another study, combination of *Bacteroides clarus* (Bc), *Fn*, Ch, and m7 showed an increase of 9 percentage points when integrated with FIT in a logistic regression model (Liang et al., 2017). In conclusion, clinical screening programs based on both microbial markers and FIT/FOBT are cost-effective and present a promising diagnostic tool.

#### Prospects and Challenges

**FIGURE 5:**
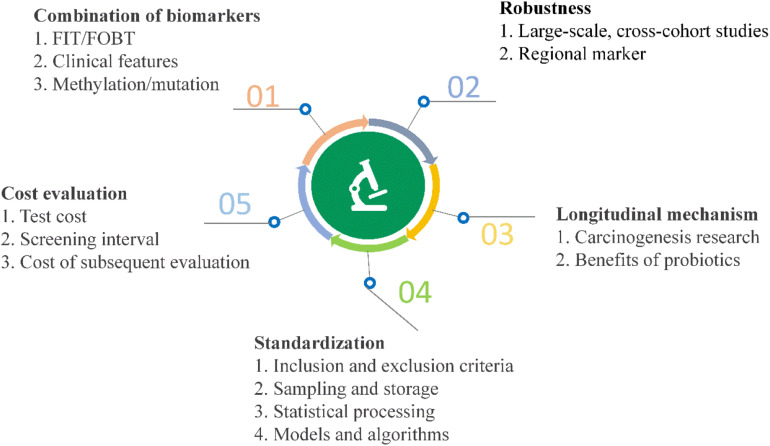
Current challenges and opportunities for early diagnosis of CRC using microbial markers.

High-throughput sequencing and other analyses over the past decade have facilitated significant advances and gradual elucidation of the role of microbes in CRC. Current research on the value of clinical transformation of microbial markers in CRC diagnosis highlights the continued challenges of using available data effectively for making a contribution to precision medicine. Inspiration from other fields may additionally facilitate novel breakthroughs (Figure 5).

Formation of CRC is a multifactorial process and potential complementary effects between molecular markers require further attention. More than 80% CRC results from chromosomal instabilities, including mutation of the adenomatous polyposis coli (APC) gene and K-ras oncogene. APC gene-deficient mice can spontaneously grow tumors in the intestine and patients carrying the KRAS mutation show chemotherapeutic resistance (Colnot et al., 2004; Kuipers et al., 2015). Fecal DNA samples have been used to detect colorectal neoplasia (Imperiale et al., 2004). Septin 9 gene methylation has been shown to be effective as a biomarker and approved by the FDA (Lofton-Day et al., 2008). Meanwhile, methylation of bone morphogenic protein 3 and N-Myc downstream-regulated gene 4 displayed high specificity as an early and frequent event in colorectal tumors (Melotte et al., 2009; Loh et al., 2010). In 2014, multitarget stool DNA testing of combined KRAS, BMP3, NDRG4, and FIT achieved significantly higher detection of cancers, which led to FDA approval of Cologuard (Imperiale et al., 2014). Therefore, integration of genomics with microbiome analysis presents a promising direction. A recent study discussed this issue, suggesting that associations between tumor genomics and the microbiome could be beneficial in diagnostics (Burns and Blekhman, 2018). Since about 11% CRC cases result from overweight and obesity, other researchers used clinical data, such as body mass index (BMI) representing overall body fat, which displayed excellent discriminatory ability. However, no statistical significance was observed in a number of other analyses (Bardou et al., 2013; Zackular et al., 2014). To extract data from plain text files, Natural Language Processing methods or software have been employed for effective use of clinical features (Yim et al., 2016). Overall, these findings offer possible solutions and important directions for future research.

Universality is another key challenge, since differing opinions exist with regard to universal microbial markers. On the one hand, cross-cohort studies and meta-analyses have provided practicable and effective strategies that could overcome heterogeneity and ethnic differences with unbiased bioinformatics and statistical analysis. For instance, an earlier metagenomics analysis involving five ethnically different cohorts identified not only known biomarkers such as Fn, Ps, Pm, and *Solobacterium moorei*, but also a novel strain, *Peptostreptococcus anaerobius*, with subsequently confirmed roles in carcinogenesis using a Apc^Min/+^ mouse model (Yu et al., 2017a; Long et al., 2019). Numerous meta-analyses also leveraged 16S rRNA or metagenomics data sets to reveal altered microbiome. Wirbel et al. (2019) identified a core set of 29 species while Dai et al. (2018) found 69 CRC-associated bacteria with metagenomic analysis. Similarly, two other teams identified 25 microbial OTUs and 12 common genera based on a random forest model using 16S rRNA sequencing datasets (Shah et al., 2018; Sze and Schloss, 2018). On the other hand, (Yang et al., 2020) proposed a strategy from a new angle, which inferred that regional biomarkers display high accuracy in specific populations. This theory was also supported by another study, which identified multiple *Fusobacterium* taxa (including *F. varium* and *F. ulcerans*) in Southern Chinese populations as disease biomarkers or targets that could be tailored according to discrepancies (Yeoh et al., 2020). Both alternative strategies provide well-powered assessments.

One of the significant challenges of clinical transformation is insufficient mechanistic analysis. While efficient computational frameworks and tools based on feature selection have been developed, machine learning requires further research (Tabib et al., 2020). Distinct from FIT/FOBT and fecal DNA tests, these semi-supervised or supervised learning methods are more like a “black box” with unclear mechanism. To date, hundreds of microorganisms have been shown to be linked with CRC, among which limited numbers have been further investigated. As a case in point, Fn was shown to be overabundant in tumor versus matched normal tissue and its potential role in CRC attracted widespread research attention (Castellarin et al., 2012; Kostic et al., 2012). Over the last few years, numerous studies have supported a role of Fn in promoting colorectal carcinogenesis through various functions such as inducing inflammatory cell infiltration, modulating E-cadherin/β-catenin signaling, activating immune cells, mediating interactions between bacteria, and binding to tumor-expressing Gal-GalNAc (Rubinstein et al., 2013, 2019; Abed et al., 2016; Yang et al., 2017). These advances have enhanced our knowledge of the potential relationships between Fn and chemoresistance, metastasis and poor prognosis (Mima et al., 2016; Yu et al., 2017b; Chen et al., 2020). Therefore, detection of Fn for early screening or exploitation of inhibitors targeting related pathways may be efficacious in clinical practice. In terms of methodological aspects, Bertrand Routy proposed a viable solution involving five steps: (1) microbial metagenomics should be standardized, (2) different “omics” analyses should be integrated, (3) the amount of cultivable microbial species should be increased, (4) non-invasive sampling methods should be combined with capsule endoscopy, and (5) Avatar mouse models should be standardized and investigated (Routy et al., 2018). Overall, longitudinal profiling of etiological and protection mechanisms of microorganisms achieves higher information richness and pave the way to take advantage of gut microbiome for diagnosis.

Development of standardized methods should also attenuate inconsistency of data. Inclusion and exclusion criteria have been gradually established, including diet, treatment, genetic background, disease history, antibiotic usage history and colonoscopy, aiming to avoid intestinal microbiota changes (O’Brien et al., 2013). During transportation and storage, a low temperature of −80°C and preservative buffer, such as RNAlater or EDTA, are effective to maintain DNA stability and integrity (Carozzi and Sani, 2013). In particular, compared to freezing for preservation, smaller technical variability was introduced without disrupting subject- and time-point specificity of the gut microbiome (Voigt et al., 2015). DNA extraction exerted the most significant effect on outcome of metagenomics analysis, highlighting the standardized DNA extraction method for human fecal samples (Costea et al., 2017). To address the complex challenges posed by large-scale studies, a protocol involving collection of microbiome samples at home and shipping to laboratories for molecular analysis was developed by Franzosa et al. (2014). Furthermore, for library preparation, PCR-free based methods were recommended to reduce PCR bias and improve assembly for accurate taxonomic assignment (Jones et al., 2015). Nevertheless, lack of standardization with regard to data access, metadata and analysis tools remain a barrier to acquisition of accurate and comparative results (Laudadio et al., 2018). Data integration and system-level modeling from multiple omics platforms is one of the most promising directions of microbiome research (Nayfach and Pollard, 2016). To improve the *status quo*, comprehensive platforms, such as MicrobiomeAnalyst and gcMeta, were recently constructed for downstream statistical analysis and functional interpretation (Dhariwal et al., 2017; Shi et al., 2019). Notably, the International Human Microbiome Standards (IHMS) project is committed to coordinate the development of standard operating procedures designed to optimize data quality and comparability in the human microbiome field. SYBR Green and probe-based qPCR are two common choices toward application, the former being more economical and the latter achieving greater accuracy for absolute quantification.

Cost-effectiveness is the ultimate challenge, including the costs of testing, screening intervals and subsequent evaluations resulting from the initial test (Dickinson et al., 2015). Due to high-cost resources, colonoscopy is not generally employed as a screening tool, except in a few countries like the United States, Germany and Austria. In low-income or middle-income countries with a low incidence of CRC, colonoscopy screening strategies may not be sufficiently cost-effective for implementation (Keum and Giovannucci, 2019). Taking FIT and Cologuard as examples, although incremental costs per additional advanced adenoma (AA) and CRC detected using colonoscopy versus FIT were £7,354 and £180,778, respectively, annual FIT reduced the colonoscopy incidence by 71% in intermediate-risk patients compared to three-yearly colonoscopy surveillance (Cross et al., 2019b). Cologuard shows superior performance for screening of AA, but carries a higher cost. In terms of the rate of screening compliance, stool DNA test is associated with higher patient acceptance owing to its simplicity. A preliminary calculation showed that combination of FIT and bacterial markers would avert up to 30% of total colonoscopies as well as save an estimated 77 million € per 100,000 participants (Malagón et al., 2019). Meanwhile, usage of residual buffer from FIT cartridges is feasible for microbiota-based analysis and could greatly ameliorate the cost (Baxter et al., 2016a; Gudra et al., 2019).

Considering the collective findings, bacteriophages, viruses, archaea and fungi will be integrated into this database as biomarkers in the future. In addition, with advances in elucidation of mechanisms and omics analyses (such as transcriptomics, proteomics, and metabolomics), corresponding function descriptions should be more systematic. Systems biology and computational biology play crucial roles in mass data integration, and machine learning-based algorithms are under development for analysis of metadata to facilitate CRC diagnosis.

## Conclusion

Development of colorectal cancer is a multifactorial process in which gut microbes play an important role. Determination of dysbiosis of microbial communities and differential patterns of abundance of microorganisms as biomarkers based on sequencing, algorithms and experimental data may aid in diagnosis and reduce morbidity and mortality. Except for a few pathogenic bacteria, the relationships between several microorganisms and colorectal cancer remain to be established, which are reflected by inconsistencies among different studies. Here, a database of CRC-related microbes was constructed using SQL and basic statistical analyses were conducted to outline biomarkers at different taxon levels. Diagnostic performance and mechanisms are discussed in detail. This type of knowledge integration is important for understanding and monitoring CRC. Moreover, this database can be used to perform inquiries and comparisons across different models and databases, contributing to further study of CRC-related microbes and promotion of cost-effective and non-invasive CRC screening strategies.

## Data Availability Statement

The original contributions presented in the study are included in the article/supplementary material, further inquiries can be directed to the corresponding author.

## Author Contributions

PC and ZZ contributed to the study design and drafted the manuscript. ZZ, SG, YaL, WM, YuL, SH, RZ, YM, KD and AS performed the statistical analysis and interpretation. All authors contributed to critical revision of the final manuscript and approved the final version of the manuscript.

## Conflict of Interest

The authors declare that the research was conducted in the absence of any commercial or financial relationships that could be construed as a potential conflict of interest.

## Footnotes

1 https://itol.embl.de/
